# Five-year mortality after acute poisoning treated in ambulances, an Emergency outpatient clinic and hospitals in Oslo

**DOI:** 10.1186/1757-7241-21-65

**Published:** 2013-08-21

**Authors:** Cathrine Lund, Mari A Bjornaas, Leiv Sandvik, Oivind Ekeberg, Dag Jacobsen, Knut E Hovda

**Affiliations:** 1 Department of Acute Medicine, Oslo University Hospital Ullevaal, Kirkeveien 166, Oslo 0450, Norway; 2 Unit of Biostatistics and Epidemiology, Oslo University Hospital Ullevaal, Kirkeveien 166, Oslo 0450, Norway; 3 National Center for NBC Medicine, Department of Acute Medicine, Oslo University Hospital Ullevaal, Kirkeveien 166, Oslo 0450, Norway

**Keywords:** Emergency medical services, Mortality, Opioids, Treatment level

## Abstract

**Background:**

The long-term mortality after prehospital treatment for acute poisoning has not been studied previously. Thus, we aimed to estimate the five-year mortality and examine the causes of death and predictors of death for all acutely poisoned patients treated in ambulances, the emergency outpatient clinic, and hospitals in Oslo during 2003–2004.

**Methods:**

A prospective cohort study included all adults (≥16 years; n=2045, median age=35 years, male=58%) who were discharged after treatment for acute poisoning in ambulances, the emergency outpatient clinic, and the four hospitals in Oslo during one year. The patients were observed until the end of 2008. Standardized mortality rates (SMRs) were calculated and multivariate Cox regression analysis was applied.

**Results:**

The study comprised 2045 patients; 686 treated in ambulances, 646 treated in the outpatient clinic, and 713 treated in hospitals. After five years, 285 (14%) patients had died (four within one week). The SMRs after ambulance, outpatient, and hospital treatment were 12 (CI 9–14), 10 (CI 8–12), and 6 (CI 5–7), respectively. The overall SMR was 9 (CI 8–10), while the SMR after opioid poisoning was 27 (CI 21–32). The most frequent cause of death was accidents (38%). In the regression analysis, opioids as the main toxic agents (HR 2.3, CI 1.6–3.0), older age (HR 1.6, CI 1.5–1.7), and male sex (HR 1.4, CI 1.1–1.9) predicted death, whereas the treatment level did not predict death.

**Conclusions:**

The patients had high mortality compared with the general population. Those treated in hospital had the lowest mortality. Opioids were the major predictor of death.

## Background

Prehospital treatment for acute poisoning is becoming more common worldwide and this is also the case in Norway. In Oslo, acute poisonings are treated at three different health care levels: the ambulance service, an emergency outpatient clinic, and hospitals. In general, poisonings with a suicidal intent or with prescription medications are treated in hospital, whereas poisonings with drugs of abuse are treated mainly in the ambulance or outpatient clinic [1]. In 2003, the majority of opioid poisonings in Oslo were discharged from the ambulance after naloxone administration [2]. Furthermore, the emergency outpatient clinic treated as many poisonings as all of the hospitals in Oslo combined. In Norway, where the health services are public, the policy is to treat patients at the lowest health care level possible without impairing treatment quality. This evaluation should also be based on knowledge of long-term risks.

Several studies have focused on subgroups of self-poisonings, such as suicide attempts [3], deliberate self-harm [4], and nonfatal drug overdoses [5]. The aim of classification is the correct evaluation of intent. However, the evaluation of intent can be difficult in patients presenting with acute poisoning because they are often comatose, unwilling to report the use of illicit drugs, and even ambivalent about their wish to die, which can complicate the evaluation. Analyzing all self-poisonings within a geographical area may make it possible to generalize from the sample to a well-defined population. Acute poisoning is a risk factor for premature death and the long-term prognosis after in-hospital treatment is poor. The mortality after hospital-treated self-poisoning is increased fourfold during the 20-year period after a poisoning incident and this enhanced mortality risk is highest during the first five years [6]. However, most acute poisoning cases are not treated as inpatients. The long-term mortality after ambulance or outpatient treatment has not been studied previously. Moreover, the long-term mortality, causes of death, and excess mortality after treatment at different health care levels have not been assessed and compared previously. Thus, it is unknown whether resources are distributed to the patients at most risk. Knowledge of the causes of death and excess mortality after treatment at different health care levels may be important for identifying targets for preventive initiatives. Moreover, knowledge of the long-term prognosis is valuable for the health care workers who assist these patients.

The aims of this study were as follows. (1) Estimating the five-year mortality rates and (2) the causes of death after treatment for acute poisoning in Oslo based on the level of care (ambulance, outpatient clinic, and hospital), and comparing their mortality rate with that of the general population. (3) Studying predictors of death during the five-year period. We wanted to know whether the five-year mortality rates would be similar if the three patient populations had comprised cases with the same age, sex, and main toxic agents.

## Methods

### Study design and setting

This was a cohort follow-up study of patients discharged after treatment for acute poisoning in an original cross-sectional multicenter study conducted from April 1, 2003 to March 31, 2004 in Oslo, which is the capital of Norway. All acute poisonings in adults (≥16 years) during one year were included consecutively [2,7]. The study was performed in the four hospitals in Oslo that treat poisoned patients, the emergency outpatient clinic, and the ambulance service.

The emergency outpatient clinic is located in central Oslo, 3.5 km from the nearest hospital, and it serves the entire city at all hours. It is similar to an emergency room, but has limited resources (e.g., gastric decontamination and intubation are not performed, and no blood gas analysis equipment is available). All patients are attended by physicians and the observation limit is 24 h. The physicians working at the outpatient clinic are general practitioners, mainly with a level of training comparable to hospital residents. In contrast to other outpatient walk-in clinics, the outpatient clinic also receives patients from ambulances. Ambulance paramedics triage patients for the outpatient clinic or hospital emergency departments. In cases of opioid overdoses, however, paramedics may administer naloxone on site without further transfer. There are no standard triage criteria. Thus, decisions are based on an evaluation of the patient’s clinical condition, knowledge of the toxic agents, and the treatment options available at the outpatient clinic. In general, stable patients likely to require observation for <24 h and not likely to require hospital treatment (e.g., with N-acetylcysteine, flumazenil, gastric decontamination, intubation, or thorough laboratory testing) are brought to the outpatient clinic. Patients with symptoms and history of gamma-hydroxybutyric acid (GHB) poisoning and patients with a possible suicidal intent are usually hospitalized. Coma is a strong triage parameter for hospital admission, unless it is caused by substances of abuse and reversed by naloxone. The current principle is to treat patients at the lowest treatment level possible while still providing adequate care. The ambulance service, the outpatient clinic, and the hospitals offer free health care and are part of the national public health care system in Norway. There are no private alternatives for acute admissions. Clinical toxicology is not a medical specialty in Norway. However, the National Poisons Information Centre is available at all hours.

### Selection of participants

All adults (≥16 years) discharged after treatment for acute poisoning in ambulances, the emergency outpatient clinic, or hospitals were included by the treating physician or paramedic [7]. Laboratory testing was not performed routinely at the outpatient clinic and never in ambulances, so inclusion was based on a clinical diagnosis of acute poisoning. Poisonings were defined as exposure to assumed toxic amounts of substances. Chronic poisonings were not included. Patients who died during treatment were not included. In cases where trauma and acute poisoning were codiagnoses, poisoning had to be the primary diagnosis or warrant independent treatment.

The patients were split into three cohorts: ambulance-, outpatient-, and hospital-treated groups. Repeat patients were only included once and their visit to the highest level of care was used as the index. This was only relevant for a few patients with repeat poisoning who received treatment at a higher treatment level. Patients who transferred from ambulance to hospital were included only in the hospital cohort. Moreover, the first episode was used as the index for patients with multiple episodes at the same treatment level, although all visits were registered.

### Methods and measurements

All evaluations were made by the treating physician or paramedic, who completed a standardized form. The main toxic agent was defined as the substance assumed to be most toxic at the level assumed to be present in the patient. This was clinically evaluated by the treating physician or paramedic based on all available information, such as statements from the patient or friends, findings at the scene, history, clinical findings, or laboratory results. The main toxic agents were split into the categories: ethanol, opioids, prescription medications, and other drugs. Opioids included prescription and illicit opioids. Prescription medications (including paracetamol and ibuprophen bought over-the-counter) consisted mainly of benzodiazepines, paracetamol, and neuroleptics. Other toxic agents mainly included illicit drugs other than opioids, such as GHB, cocaine, and ecstasy. Additional toxic substances were only registered in hospitals and were not controlled for in the analyses. Consciousness was registered using the Glasgow Coma Scale (GCS) [8]. Somnolence was classified as GCS < 14 and coma as GCS < 8.

### Outcomes

At the end of the observation period (December 31, 2008), the status of each patient (alive, emigrated, or dead) was checked against the National Population Register by performing a retrospective review of the database. This is the official registry for the inhabitants of Norway, which is used by government officials. The registry includes each person’s social security number, emigration or immigration date, and date of birth and death. Deaths outside Norway are also registered. The information in the National Population Registry is confidential so we had to order the data through Statistics Norway, which is a government-owned company that oversees several registries. Trained abstractors extracted the data by performing an electronic search using the social security numbers of the study patients. They provided the status of each patient, including the date of emigration and date of death if applicable. Only patients with a permanent or temporary social security number could be traced. The others were excluded from the analyses. It may take some time before the registry is updated if a person dies or moves because Statistics Norway only releases annual data for statistical purposes after two years.

The causes of death were obtained from the National Cause of Death Registry, which is based on the official death certificates. Statistics Norway also extracted this data by performing an electronic search using the patients’ social security numbers. These death certificates provided information about one main cause of death and up to five additional causes, which were all classified according to the tenth revision of the International Classification of Diseases (ICD-10). In cases where at least one of the additional causes of death was poisoning or drug abuse, the death was considered drug-related.

### Primary data analysis

The patients were observed until death, emigration, or the end of the observation period (December 31, 2008). Based on this data, the number of studied person-years was calculated for each sex and five-year age group. The standardized mortality rates (SMRs) of the three cohorts were calculated by taking the ratio of observed to expected deaths using the national mortality rates from Statistics Norway as a reference. They provided the number of deaths per 1000 inhabitants in Norway, according to sex and five-year age groups for each year. These mortality rates were used to calculate the number of deaths that would be expected in the period 2004–2008 for a group with the same age and sex distribution as that found in the study cohorts. The 95% confidence intervals (CI) of each SMR were computed using the formula: 95% CI = (SMR/e^(1.96/√dead)^) – (SMR × e^(1.96/√dead)^) [9].

Predictors of death were identified using multivariate Cox regression analysis. The outcome variable was death. Based on previous research, the following variables were considered clinically relevant and included in the analysis: consciousness, main toxic agent, level of care, age, and sex. Consciousness was analyzed using three categories: awake, somnolent, and comatose. The main toxic agent was analyzed using four categories: ethanol, opioids, prescription medication, and other. Ethanol was used as the reference because this was the biggest group. The level of care was analyzed using three categories: hospital, outpatient clinic, and ambulance, where hospital was used as the reference. Sex was analyzed as a categorical variable. Age was divided by ten and analyzed as a continuous variable. All variables were included in the multivariate analysis, which was performed stepwise backward. The assumptions underlying multivariate Cox regression analysis were checked and satisfied. A Kaplan–Meier plot was used to illustrate the cumulative proportion of deaths over time. SPSS software (v. 19.0; IBM, Armonk, NY, USA) was used to analyze the data.

## Results

### Characteristics of study subjects

Of the 2298 patients with acute poisoning during the inclusion period, we excluded 236 patients with unknown social security numbers, 15 patients who died during the index episode, and two patients with residency outside Norway. Thus, the number of patients included was 2045. The median age was 35 years (range 16–92 years, interquartile range 26–46) and 58% were males (Table 1).

**Table 1:** Description of the 2045 patients treated for acute poisoning in Oslo in 2003 at the time of admission

	**Ambulance**		**Outpatient clinic**		**Hospital**	
	**N**	**(%)**	**N**	**(%)**	**N**	**(%)**
Median age (yrs)	35		36		35	
Sex						
- Males	445	(65)	434	(67)	312	(44)
Main toxic agent						
- Ethanol	233	(34)	344	(53)	115	(16)
- Prescription drugs	69	(10)	92	(14)	412	(58)
- Opioids	324	(47)	128	(20)	51	(7)
- Other^1^	60	(9)	82	(13)	135	(19)
Consciousness						
- Awake	350	(51)	228	(35)	354	(49)
- Somnolent	137	(20)	344	(53)	196	(28)
- Comatose	199	(29)	74	(12)	163	(23)
Total	686	(100)	646	(100)	713^2^	(100)

^1^Other included mainly illicit drugs other than opioids, such as gamma-hydroxybutyrate and cocaine.

^2^Of these, 416 patients had polysubstance ingestions.

### Main results

**Figure 1:**
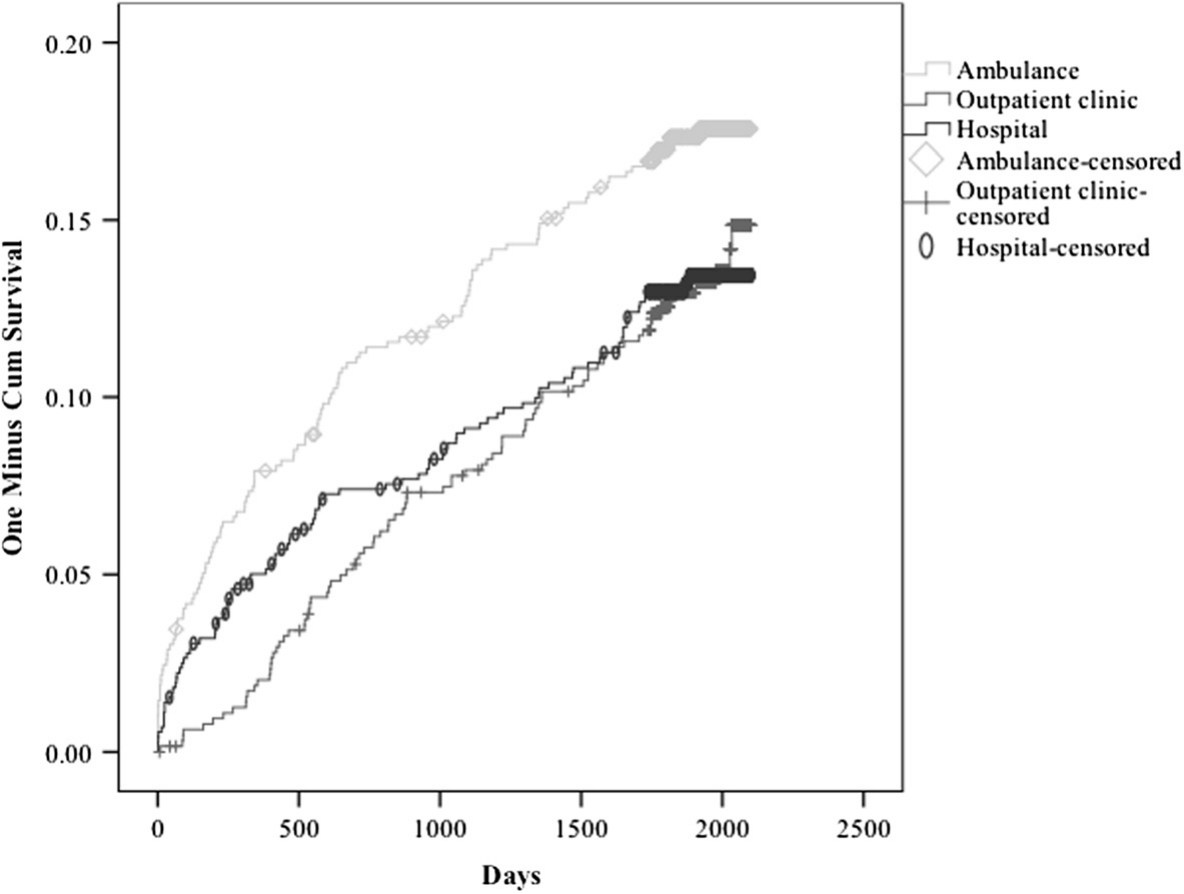
**Cummulative proportion of deaths after ambulance, outpatient and hospital treatment of acute poisoning in Oslo in 2003.** Kaplan Meier plot illustrating the cummulative proportion of deaths after treatment of acute poisoning in hospitals, the Emergency outpatient clinic and ambulances in Oslo during the five-year follow-up period.

During the five-year follow-up, 285 patients died, i.e., 112 (16%) of those treated in ambulances, 86 (13%) of those treated in the emergency outpatient clinic, and 87 (12%) of those treated in hospitals (Table 2). According to the National Registry, the expected number of deaths for a matched population would have been 33; ten of those treated in ambulances, nine of those treated in the emergency outpatient clinic and 15 of those treated in hospitals. The median age for those who died was 47 years (IQR 35–67). Four patients died within the first week. The Kaplan–Meier plot detected a slightly higher overall mortality during the first year whereas there was an almost linear mortality plot throughout the five-year period (Figure 1). The overall SMR was 9 (95% confidence interval 8–10); 12 (CI 9–14) for those treated in ambulances, 10 (CI 8–12) for those treated in the outpatient clinic, and 6 (CI 5–7) for those treated in hospitals. There was no difference in SMRs for males and females. In each cohort, SMR was highest among patients admitted for opioid poisoning. For the opioid poisoning patients, SMR was lowest among those treated in hospitals.

**Table 2:** Five-year excess mortality according to sex and main toxic agent

		**Ambulance**		**Outpatient clinic**		**Hospital**		**Total**	
Sex									
- Males	Dead N (%)	85	(19)	68	(16)	43	(14)	196	(16)
	SMR (CI)	12.7	(10.0–15.5)	9.5	(7.5–12.1)	5.5	(3.9–7.2)	9.1	(7.8–10.3)
- Females	Dead N (%)	27	(11)	18	(9)	44	(11)	89	(10)
	SMR (CI)	9.0	(5.6–12.4)	11.5	(6.2–16.8)	6.3	(4.4–8.1)	7.7	(6.1–9.3)
Main toxic agent									
- Opioid	Dead N (%)	57	(18)	23	(18)	12	(24)	92	(18)
	SMR (CI)	35.1	26.0–44.2)	35.3	(23.5–53.0)	10.4	(4.5–16.2)	26.8	(21.3–32.3)
- Ethanol	Dead N (%)	32	(14)	50	(15)	18	(16)	100	(14)
	SMR (CI)	5.5	(4.3–7.0)	7.8	(5.9–10.3)	8.1	(4.4–11.9)	6.9	(5.6–8.3)
- Prescription drugs	Dead N (%)	14	(20)	4	(5)	48	(12)	66	(12)
	SMR (CI)	9.0	(5.3–15.2)	6.5	(2.4–17.3)	5.1	(3.6–6.5)	5.7	(4.3–7.0)
- Other	Dead N (%)	9	(15)	9	(10)	9	(7)	27	(10)
	SMR (CI)	13.6	(4.7–22.5)	8.7	(4.5–16.7)	4.6	(1.6–7.6)	7.4	(4.6–10.2)
Total	Dead N (%)	112	(16)	86	(13)	87	(12)	285	(14)
	SMR (CI)	11.6	(9.4–13.7)	9.9	(8.0–12.2)	5.9	(4.6–7.1)	8.6	(7.6–9.6)

SMR: Age and sex adjusted standardized mortality rate.

Excess mortality in the 2045 patients treated for acute poisoning at different health care levels in Oslo in 2003.

During the five-year study period, 101 (5%) patients died in accidents, 44 (2%) from cardiovascular events, 32 (2%) by suicide, 27 (1%) from cancer, and 81 (4%) from other causes (Table 3). Among the accidental deaths, 66 (65%) had poisoning or substance dependence as an underlying death diagnosis. Moreover, 20 of the patients who died from other causes had alcohol or opioid dependence as an underlying cause of death. Among the suicides, 53% died of poisoning, 31% by hanging, and 16% by drowning, jumping from a height, or in intentional car crashes. SMR for accidental death was highest among those treated by ambulance (SMR 36, CI 27–46), while SMR for suicide was highest among those treated in hospitals (SMR 12, CI 8–19) (Table 4).

**Table 3:** Causes of death by treatment level and sex

	**Ambulance**				**Outpatient clinic**				**Hospital**				**Total**			
	**Males**		**Total**		**Males**		**Total**		**Males**		**Total**		**Males**		**Total**	
	**N**	**(%)**	**N**	**(%)**	**N**	**(%)**	**N**	**(%)**	**N**	**(%)**	**N**	**(%)**	**N**	**(%)**	**N**	**(%)**
Accident	40	(9)	54	(8)	22	(5)	28	(4)	11	(4)	19	(3)	73	(7)	101	(5)
Cardiovascular	14	(3)	17	(2)	12	(3)	14	(2)	9	(3)	13	(2)	34	(3)	44	(2)
Suicide	5	(1)	7	(1)	2	(<0.5)	6	(1)	7	(2)	19	(3)	14	(1)	32	(2)
Cancer	6	(1)	9	(1)	7	(2)	10	(2)	5	(2)	8	(1)	18	(1)	27	(1)
Other	20	(4)	25	(4)	25	(6)	28	(4)	11	(3)	28	(4)	56	(5)	81	(4)
Total	85	(19)	112	(16)	68	(16)	86	(13)	43	(14)	87	(12)	196	(17)	285	(14)

Among the suicides, 17 (53%) were by poisoning and 10 (31%) were by hanging.

Other included 20 patients dying of alcohol or opioid dependence.

Causes of death in 2045 patients treated for acute poisoning in Oslo in 2003.

**Table 4:** Excess mortality for different causes of death

	**Ambulance**		**Outpatient**		**Hospital**		**Total**	
	**SMR**	**CI**	**SMR**	**CI**	**SMR**	**CI**	**SMR**	**CI**
Cardiovascular	3.6	(2.2–5.8)	3.1	(1.8–5.2)	1.9	(0.8–2.9)	2.7	(1.9–3.5)
Cancer	1.2	(0.6–2.3)	1.4	(0.8–2.6)	0.9	(0.5–1.8)	1.2	(0.8–1.7)
Suicide	4.6	(2.2–9.6)	3.9	(1.7–8.7)	11.8	(7.5–18.5)	6.8	(4.8–9.6)
Accident	36.1	(26.5–45.7)	18.9	(13.0–27.4)	15.2	(8.4–22.0)	23.9	(19.2–28.6)
Total	11.6	(9.4–13.7)	9.9	(8.0–12.2)	5.9	(4.6–7.1)	8.6	(7.6–9.6)

SMR: Age and sex adjusted standardized mortality rate.

SMRs was not calculated for the 80 patients dying of other causes.

Excess mortality among the 2045 patients treated for acute poisoning at different health care levels in Oslo in 2003.

A multivariate Cox regression analysis of all patients was performed to determine the predictors for death and to test whether the mortality would have been similar if the three groups had been matched in terms of age, sex, and main toxic agent. Factors at the time of inclusion that predicted death were opioids as main agent (HR 2.3, CI 1.6–3.0), older age (HR 1.6, CI 1.5–1.7), and male sex (HR 1.4, CI 1.1–1.9) (Table 5). The level of care was not associated with death in the adjusted analysis.

**Table 5:** Factors predicting death during the five-year follow-up

	**N**	**Dead (N)**	**HR**	**CI**
Age (+10 years)			1.6	(1.5–1.7)
Sex				
- Females	854	89	Ref	
- Males	1191	196	1.4	(1.1–1.9)
Main toxic agent				
- Ethanol	692	100	Ref	
- Prescription drugs	573	66	1.1	(0.8–1.5)
- Opioids	503	92	2.3	(1.6–3.0)
- Other	277	27	1.1	(0.7–1.7)
HLC				
- Hospital	713	87	Ref	
- Outpatient clinic	646	86	1.1	(0.8–1.5)
- Ambulance	686	112	1.1	(0.8–1.6)

CI: Confidence interval, HLC: Highest level of care, HR: Hazard ratio.

Results of multivariate Cox regression analysis of the 2045 patients treated for an acute poisoning in Oslo at different health care levels in 2003.

## Discussion

One in seven patients died during the five-year study period. Given the low median age of this patient population, this mortality rate was extremely high. The expected number of deaths would have been one out of 100 patients. The SMR would have been slightly higher if we had included the patients who died during treatment. However, most of these patients were announced dead upon arrival, or within one hour of admission. The five-year mortality rates were high in all three cohorts. To the best of our knowledge, no other studies have assessed the long-term mortality after prehospital treatment of acute poisoning. The overall excess mortality of 9 found in our study is higher than the ten-year SMR of 6 after a suicide attempt by poisoning reported in a Danish study by Nordentoft et al. [10], and the 12-year SMR of 7 among opioid addicts reported in an American study by Joe et al. [11]. The SMR after hospital treatment was similar to the five-year mortality in a previous hospital study conducted in Oslo during 1980 (SMR 6, CI 5–7 vs SMR 6, CI 5–8) [6]. The mortality rate has remained relatively stable.

After correcting for the expected number versus the observed number of deaths, the SMRs had no sex differences. In terms of absolute numbers, males had a higher mortality ratio than females, but the expected number of deaths among males was also higher. Therefore, the SMRs were similar for males and females. The overall mortality increased gradually with age, but so did the expected number of deaths. As such, age had no significantly effect on the SMR. The mortality was particularly high among those treated for opioid poisonings (SMR 29). Although they were not identical cohorts, the SMRs among those poisoned by opioids were similar to those of intravenous drug users in Glasgow, Scotland (SMR 22) and heroin addicts in Catalonia, Spain (SMR 29) [12,13]. A higher proportion of the patients treated in hospital for opioid poisoning died, but the SMR was lower in this cohort, which reflected their older age. It may also reflect the fact that a higher proportion of poisonings with prescription opioids are treated in hospital whereas intravenous heroin overdoses are predominantly treated in a prehospital setting [2,7].

The overall SMR in our study was lowest among patients treated in hospitals. This was explained partly by the higher proportion of opioid poisonings treated in prehospital settings. Four out of five patients treated in hospital were referred to follow-up compared with none of those treated by the ambulance service, which may have had a protective effect [2,14]. However, the patients treated in hospitals were probably more severely poisoned. Thus, it is unknown whether the mortality would have been higher if they had been treated in a prehospital setting.

Suicides and overdoses were the major concerns in the present patients. Our results were compared with the national figures provided by Statistics Norway so the deaths related to substance dependence had to be included among “other deaths” (chapter F in ICD-10). Accidents, mainly drug-related, were the major cause of death in each cohort. The suicide methods used by females in this cohort reflected the national figures, where poisonings were most common, followed by hanging. However, the low number of suicides (n = 32) made this evaluation difficult. The high risk of accidental death (24 times that of the general population) may indicate that these patients have a high-risk lifestyle both in terms of drug abuse and risk of trauma. We also detected a threefold increase in deaths due to cardiovascular diseases. Intravenous substance abuse is a risk factor for infective endocarditis, which may have contributed partly to this excess mortality [15]. Furthermore, there is an increased risk of acute coronary syndrome and arrhythmias with stimulant use, especially cocaine and amphetamines [16,17]. The risk of cardiovascular disease is also higher in lower socioeconomic groups into which most drug abusers are classified [18].

The lower five-year mortality among hospital-treated acute poisonings might suggest that hospital treatment is safer but this hypothesis was not supported after adjusting for age, sex, and main toxic agent. The major predictor for death was opioid poisoning. This adjusted result implies that the mortality would not be different if the three groups had been similar in terms of age, sex, and main toxic agent. Therefore, the results do not indicate that patients would have a better outcome if they were treated in hospital. However, they do suggest that the patients treated in the prehospital setting are a high-risk group who require special attention. Most deaths occurred more than one week after discharge. Thus, avoiding repetition might be an important step in reducing the high mortality rate. Ambulance records state that several patients rejected further transport when it was offered. No follow-up was initiated by ambulance personnel and only half of the patients treated in the outpatient clinic were referred to follow-up. Therefore, hospitalization might be beneficial to ensure the initiation of follow-up. However, the motivation among these patients may be a challenge.

The mortality in this group was high compared with the general population, which is a challenge to our society. It is important to remember the poor long-term prognosis of these patients. The excess mortality after opioid overdose is of particular concern and it is possible that more resources should be focused on this particular group. More follow-ups should be considered, but these patients are difficult to follow up and the evidence of its effects is limited. Indeed, few studies have demonstrated reduced mortality after follow-up treatment. Sorensen et al. found a decrease in mortality among opioid abusers achieving abstinence [19], whereas Bjornaas et al. found no difference in the mortality of patients referred to voluntary detoxification [20]. Moreover, Haastrup et al. found that death rather than abstinence was the main contributor to the declining number of drug abusers with age among patients who received drug addiction treatment [21]. However, offering follow-up after discharge from the prehospital setting, such as providing a phone number, may be a simple but important step towards motivating for treatment and lowering the mortality.

### Limitations

Patients were triaged according to normal procedure for ethical reasons. As such, this was no randomized trial and the results must be interpreted accordingly. Unfortunately, we were unable to trace the status of each patient at discharge retrospectively and we could only do so for the groups as a whole. Thus, patients may have repeated their poisoning and received treatment at a higher treatment level after the end of the inclusion year. For practical reasons, all patients were observed until the 31st of December 2008, regardless of the date of inclusion. As such, some patients were observed for less than five years. No registry is without faults but the National Population Registry provided the official and best data that could be obtained. Therefore, missed deaths or missed emigrations are unlikely to be important sources of bias. The data extraction was performed electronically by an independent abstractor, which minimized measurement errors and interrater variability. The assessment of toxic agents may have been less precise in the prehospital setting due to limited diagnostic resources. It is debatable whether a more thorough verification of toxic agents should have been performed routinely. However, treatment is mainly symptomatic and not based on the laboratory detection of toxic agents. Therefore, this study was based on the clinical findings and blood/urine tests used in routine clinical settings, which makes the results generalizable. Furthermore, the limited value of laboratory results has been demonstrated in clinical settings [22,23]. We did not assess the interrater variability of the evaluation of main toxic agents. Unfortunately, we did not register basic health status because the original form was short to ensure its feasibility. Registering suicidal intent and socioeconomic data may have provided important additional information, especially in the regression analysis. Unfortunately, this could not be performed in prehospital settings. A postmortem evaluation of suicide can be challenging, so there may have been unregistered suicides among the accidental deaths. Furthermore, the mortality rates in the cohorts were compared with national rates and not adjusted for socioeconomic status. However, socioeconomic differences are relatively small in Norway.

## Conclusions

In summary, the five-year mortality rate was considerably higher than that among the general population. The patients treated in ambulances and the outpatient clinic had a higher mortality than those treated in hospital. This was explained partly by the high proportion of opioid poisonings treated in the prehospital setting. Patients treated for opioid poisoning had a particularly high excess mortality rate and opioid poisoning was the major predictor for death. The main cause of death in all three cohorts was accidents, which were mainly drug-related.

## Abbreviations

CI: Confidence interval; GCS: Glasgow Coma Scale; HLC: Highest level of care; HR: Hazard ratio; ICD: International Classification of Diseases; SMR: Standardized mortality rate.

## Competing interests

The authors declare that they have no competing interests.

## Authors’ contributions

MAB, KEH, OE and DJ conceived the study and designed the trial. MAB and CL obtained and managed the data from the National Death Register. CL performed the statistical analysis. LS provided statistical advice and controlled the statistical analysis. CL drafted the manuscript and all authors contributed substantially to its revision. All authors take the responsibility for the paper as whole. All authors read and approved the final manuscript.
